# Cartilage-hair hypoplasia–anauxetic dysplasia spectrum disorders harboring *RMRP* mutations in two Korean children: A case report

**DOI:** 10.1097/MD.0000000000037247

**Published:** 2024-05-24

**Authors:** Ju Heon Park, Minji Im, Yae-Jean Kim, Ja-Hyun Jang, Sae-Mi Lee, Min-Sun Kim, Sung Yoon Cho

**Affiliations:** a Department of Medicine, Sungkyunkwan University School of Medicine, Seoul, Korea; b Department of Pediatrics, Samsung Medical Center, Sungkyunkwan University School of Medicine, Seoul, Korea; c Department of Laboratory Medicine and Genetics, Samsung Medical Center, Sungkyunkwan University School of Medicine, Seoul, Korea; d Green Cross Genome, Yongin, Korea.

**Keywords:** cartilage-hair hypoplasia, immunodeficiency, RMRP, short stature, skeletal dysplasia

## Abstract

**Rationale:**

Cartilage-hair hypoplasia (CHH, OMIM # 250250) is a rare autosomal recessive disorder, which includes cartilage-hair hypoplasia–anauxetic dysplasia (CHH-AD) spectrum disorders. CHH-AD is caused by homozygous or compound heterozygous mutations in the RNA component of the mitochondrial RNA-processing Endoribonuclease (*RMRP*) gene.

**Patient concerns:**

Here, we report 2 cases of Korean children with CHH-AD.

**Diagnoses:**

In the first case, the patient had metaphyseal dysplasia without hypotrichosis, diagnosed by whole exome sequencing (WES), and exhibited only skeletal dysplasia and lacked extraskeletal manifestations, such as hair hypoplasia and immunodeficiency. In the second case, the patient had skeletal dysplasia, hair hypoplasia, and immunodeficiency, which were identified by WES.

**Interventions:**

The second case is the first CHH reported in Korea. The patients in both cases received regular immune and lung function checkups.

**Outcomes:**

Our cases suggest that children with extremely short stature from birth, with or without extraskeletal manifestations, should include CHH-AD as a differential diagnosis.

**Lessons subsections:**

Clinical suspicion is the most important and *RMRP* sequencing should be considered for the diagnosis of CHH-AD.

## 1. Introduction

Cartilage-hair hypoplasia–anauxetic dysplasia (CHH-AD) spectrum disorder is an encompassing diagnosis that includes metaphyseal dysplasia without hypotrichosis (MDWH, OMIM #250460), CHH (OMIM # 250250), and AD (OMIM #607095). Over 90 *RMRP* variants have been identified in CHH-AD spectrum disorders.^[1]^ Affected individuals with CHH-AD have been reported in most populations, but especially high incidences were found in Finland and the Amish populations, with a prevalence of 1:23,000 (carrier frequency of 1:76) and 1 to 2:1000 (carrier frequency of 1:10), respectively, and about 700 individuals are currently reported having CHH.^[2]^ CHH is an autosomal recessive disorder caused by a homozygous or compound heterozygous mutation in the RNA component of the mitochondrial RNA-processing Endoribonuclease (*RMRP*) gene on chromosome 9p13.^[3]^

CHH is characterized by metaphyseal chondrodysplasia with disproportionate short stature (reported adult heights range from 104 to 149 cm), fine and sparse hair, ligamentous laxity, immunodeficiency, hypoplastic anemia, cancer predisposition, neuronal dysplasia of the intestine (including congenital megacolon), and normal intelligence.^[2]^ Short, thick long bones, metaphyseal flaring, and irregularities with globular epiphyzes at the knees and ankles are typical radiologic features of CHH.^[4]^ Immunodeficiency of CHH can present as either T-cell or B-cell deficiencies and can manifest in infancy as severe combined immunodeficiency (SCID) or slowly progress and manifest in late adolescence/adulthood.^[3,5]^ Anemia, usually hypoplastic anemia, is also seen in over 80% of CHH patients and is usually mild and self-limited; however, some patients demonstrate severe, persistent anemia.^[6]^ MDWH cases only present as metaphyseal chondrodysplasia with disproportionate short stature without extraskeletal manifestation, such as defective immunity or hypoplastic hair. The global incidence of MDWH is unknown; to date, only 20 MDWH patients have been confirmed by *RMRP* analysis.^[4–7]^ AD, described as a form of spondylo-meta-epiphyseal dysplasia, is characterized by the prenatal onset of extreme short stature (adult heights <85 cm), hypodontia, and mild mental retardation.^[7,8]^

A diagnosis of a CHH-AD spectrum disorder is established by the clinical and characteristic radiologic findings. Following this, the identification of biallelic pathogenic or likely pathogenic variants in the *RMRP* gene by molecular genetic testing can confirm the diagnosis, and the patient can be considered for family studies.^[9]^

To the best of our knowledge, there are no previous reports of CHH in Korea. In the present study, we describe 2 cases of Korean patients with CHH-AD, 1 of which is the first case of CHH in Korea. We report their clinical, radiographic, and diagnostic processes with genetic testing.

## 2. Case report

### 2.1. Case 1

A 13-month-old male visited our clinic owing to his short stature. The patient was born at the gestational age of 40 weeks with a birth height of 46 cm (z-score calculated by the 2017 Korean National Growth Chart for children, −2.04 standard deviation score [SDS]) and birth weight of 3040 g (−0.55 SDS) from nonconsanguineous healthy Korean parents. The patient had a younger male sibling of normal height and showed no skeletal dysplasia. At the time of visit, the patient’s height was 61.4 cm (−6.47 SDS), and weight was 6.1 kg (−3.35 SDS). The mid-parental height (paternal height, 180 cm; maternal height, 159 cm) was 176 cm. The patient had no history of recurrent infections or congenital megacolon. The patient did not have joint hypermobility, facial anomaly, hypodontia, hypotrichosis, or hepatosplenomegaly. The patient had 1 Mongolian spot on the posterior lower trunk, the only cutaneous finding. The patient had a rhizomelic short stature, brachydactyly, bowing of the tibia, genu varum, short feet, and kyphosis. The patient had normal development milestones.

**Figure 1.**
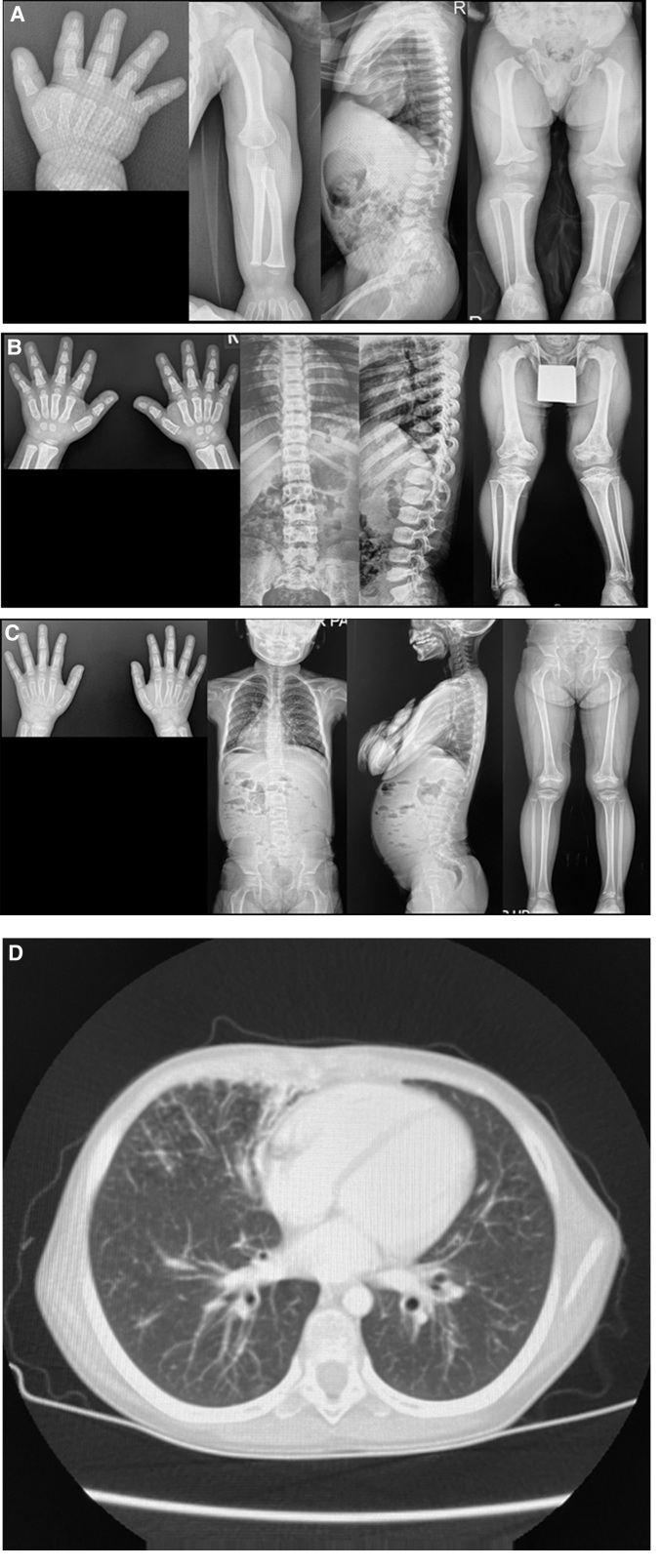
Skeletal survey of both patients and chest computed tomography (CT) of case 2. (A) Radiographs of the patient in case 1, aged 13 mo; both hands and distal forearms show metaphyseal irregularities and sclerosis of the distal radius and ulnae and mildly cone-shaped epiphyzes of the phalanges. They also show kyphosis and genu varum. (B) Radiographs of the patient in case 1, aged 8 yr and 2 mo. (C) Radiographs of the patient in case 2, aged 8 yr and 9 mo; both knees and hips show metaphyseal irregularities and sclerosis. (D) Chest CT of the patient in case 2 aged 10 yr and 1 mo.

The patient’s radiographic features revealed subtle cupping and widening at the metaphysis of both metacarpal bones, phalanges, and ulnae, as well as bilateral acetabulum dysplasia, femur shortening (rhizomelia), and brachy-phalanges. The chest X-ray revealed a normal thymus (Fig. 1). The complete blood count was normal, but the neutrophil ratio was low at 35.0% (reference range [RR], 41.5%–73.5%) and the lymphocyte ratio was high at 54.0% (RR, 19.9%–49.2%). The electrolytes, renal function, liver enzymes, and alkaline phosphatase were within the normal ranges. The thyroid, adrenal function test, and insulin-like growth factor-1 (IGF-1) levels were normal. The sella MRI and 2D echocardiography were normal. The patient was suspected of having skeletal dysplasia, and a growth hormone (GH) stimulation test was not done. A GH treatment was not tried.

At the age of 7 years and 4 months, the patient revisited our clinic due to progressive delayed growth. The patient’s height was 87 cm (−7.21 SDS), and his weight was 13.5 kg (−2.65 SDS). Whole exome sequencing (WES) was performed to investigate the genetic cause of the patient. After obtaining written informed consent, genomic DNA was extracted from peripheral blood; a cDNA library was prepared using a TruSight One Sequencing Panel (Illumina, Inc., San Diego, CA), which enriched a 12-Mb region spanning 62,000 target exons from a total of 4813 clinically relevant genes. Massively parallel sequencing was achieved on an Illumina NextSeq platform. Sequence reads were mapped to the UCSC hg19 standard base for conducting a comparative analysis.

**Figure 2.**
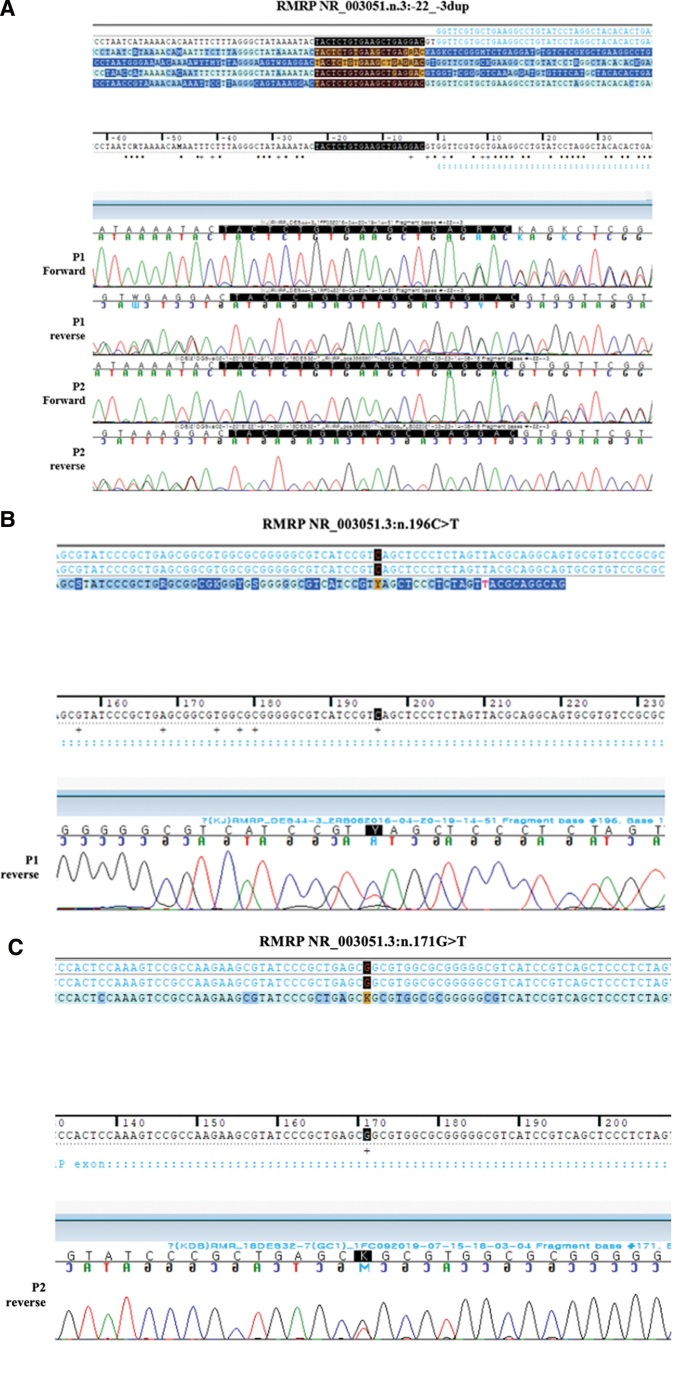
Chromatograms of case 1 and case 2. (A) Chromatogram of *RMRP* NR_003051.3:n.-22_-3dup. P1: the patient in case 1; P2: the patient in case 2. (B) Chromatogram of *RMRP* NR_003051.3:n.196C > T in P1: the patient in case 1. (C) Chromatogram of *RMRP* NR_003051.3:n.171G > T in P2: the patient in case 2. RMRP = the RNA component of the mitochondrial RNA-processing Endoribonuclease.

The WES results revealed 2 variants in the *RMRP* gene (NR 003051.3:n.-22-3dup and NR 003051.3:n.196C > T). The patient’s father and male sibling have also been confirmed to carry the NR 003051.3:n.-22 -3dup, a known pathogenic variant, while the patient’s mother carries the NR 003051.3:n.196C > T, a known pathogenic variant (Fig. 2). Consequently, these 2 variants were confirmed to be compound heterozygous, and the patient was diagnosed with MDWH. At the time, the patient had a negative immunodeficiency test, and regular immunology tests have been performed. Since the diagnosis, in the outpatient clinic, the patient is continuously tracking whether symptoms have occurred. At the time of 15 years and 4 months, the patient was 103.3 cm (−11.63 SDS) tall and weighed 28.8 kg (−3.20 SDS), showing no evidence of immunodeficiency or hair hypogenesis. Long-term follow-ups are planned to determine whether CHH-related symptoms have occurred.

### 2.2. Case 2

An 8-years 9-months-old female was referred to our clinic to evaluate her short stature and frequent episodes of infections. The patient was born at the gestational age of 40 weeks with a birth weight of 2700 g (−1.14 SDS) from nonconsanguineous healthy Korean parents. At the time of the visit, the patient’s height was 99.9 cm (−5.45 SDS), and her weight was 16.3 kg (−2.21 SDS). The mid-parental height (paternal height, 178 cm; maternal height, 163 cm) was 164 cm. The patient suffered from recurrent pneumonia (1–2 times per year) and watery diarrhea. The patient had erythematous patches around her right elbow, and both knees and heels. The patient had thin hair and showed no evidence of breast development or other secondary sexual characteristics. The patient had normal psychomotor development. The patient’s karyotype result was 46, XX, and her bone age was 1 year and 8 months younger than her chronologic age. The patient has a 4-year-old healthy younger female sibling, and 1 died younger male sibling died from pneumonia with neutropenia at 18 months old.

The skeletal survey demonstrated metaphyseal striation with irregular contour around both knees, genu varum, and kyphosis. In addition, the skeletal survey showed compression fractures of both ankles and the lumbar spine (Fig. 1). The laboratory tests revealed that her hemoglobin level was 11.1 g/dL (RR, 11.2–14.8 g/dL), leukocyte count was 7270/μL (RR, 3150–8630/μL), absolute neutrophil count was 5850/μL (RR, 1570–8300/μL) and absolute lymphocyte count was 930/μL (RR, 1000–4800/μL). However, the neutrophil ratio was 80.4% (RR, 40.6%–73.5%) and the lymphocyte ratio was 56.0% (RR, 20.0%–50.8%). The serum calcium level was 9.1 mg/dL (RR, 8.6–10.2 mg/dL), phosphate level was 5.1 mg/dL (RR, 2.5–4.5 mg/dL), and alkaline phosphatase level was 99 U/L (RR, 35–104 U/L). The aspartate aminotransferase (AST) and alanine aminotransferase (ALT) levels were 440 U/L (RR, 0–32 U/L) and 557 U/L (RR, 0–33 U/L). A follow-up test 3 months later showed an AST of 66 U/L and an ALT of 65 U/L without any treatment, indicating that the elevated liver function tests were due to a temporary viral infection. The IGF-1 level was low at 28.9 ng/mL (RR, 93.5–441.3 ng/mL), and the GH stimulation test showed a normal response (>10 μg/L). The thyroid and adrenal function tests were normal, and a skin biopsy for erythematous patches revealed scattered noncaseating granuloma with a negative finding on acid-fast bacillus stain, indicative of a possible infection. The sella turcica and Parasellar region magnetic resonance imaging were normal, but pansinusitis and bilateral mastoid effusion were incidentally found.

At 10 years and 1 month of age, the patient came to our dermatology clinic because of the uncontrolled warts that they acquired from her female sibling. At that time, fine, sparse hair was detected. A WES was performed and revealed 2 variants in the *RMRP* gene, confirmed by Sanger sequencing, NR_003051.3:n.-22_-3dup (likely pathogenic variant) and NR_003051.3:n.171G > T (variant of uncertain significance), and the patient was diagnosed with CHH (Fig. 2). Parental genetic testing was impossible since neither parent consented to genetic testing.

Screening tests for immunodeficiency revealed both cellular and humoral immunodeficiencies. The results of a lymphocyte subset analysis revealed a T-cell count of 373/μL (RR, 700–4200/μL), CD 4 + helper T-cell count of 192/μL (RR, 300–2000/μL), CD 8 + helper T-cell count of 142/μL (RR, 300–1800/μL), B-cell count of 47/μL (RR, 200–1600/μL), and NK cell count of 377/μL (RR, 90–900/μL). However, the results of immunoglobulin tests, including IgG, IgA, IgM and IgE, were all within normal ranges. The patient had a hemoptysis at the age of 11 years and 6 months, and a chest computed tomography revealed that the patient had postinfectious bronchiolitis obliterans with bronchiectasis (Fig. 1). The patient received regular immune and lung function checkups, and her last visit was when she was 11 years and 10 months old. Since the patient has been lost to follow-ups for 2 years and 4 months, her survival is unknown.

## 3. Discussion

This report describes 2 cases of CHH-AD spectrum disorders with *RMRP* variants (Table 1). Two patients had extremely short statures (below −4 SDS) on presentation. Knowing the birth length is an important prognostic factor for the mortality of patients with CHH. Vakkilainen et al^[10]^ reported that in patients with a birth length adjusted for gestational age below −4 SDS, mortality, which is directly related to immune dysfunction, was significantly higher. The patient in case 1 had a low birth height, but it was not below −4 SDS, and the patient in case 2 lacked birth data. Skeletal dysplasia was present in both cases, but the radiographic findings of case 1 were subtle, whereas those of case 2 were typical. In case 1, bilateral acetabulum dysplasia, femur shortening (rhizomelia), and brachy-phalanges were the only findings of the skeletal survey at the age of 13 months. Although metaphyseal changes are the most distinctive feature of CHH, radiographic findings, including metaphyseal changes, in infants can be subtle.^[11,12]^ In case 2, the skeletal survey demonstrated metaphyseal striation with irregular contour around both knees and ankles, as well as a compression fracture of the lumbar spine, which are typical findings in CHH.

**Table 1:** Genotype and phenotype of the patients.

Patient	Age at diagnosis	Sex	Genotype		Phenotype						
	(yr/mo)		Allele 1	Allele 2	BH (SDS)	BW (SDS)	HF (SDS)	SD	HH	ID	Others
Case 1*	1/1	M	-22_-3dup‡	n.196C > T§	46 cm (−2.04)	3040 g (−0.55)	61.4 cm (−6.47)	+	−	−	None
Case 2†	8/9	F	-22_-3dup	n.171G > T	N/A	2700 g (−1.14)	99.9 cm (−5.45)	+	+	+	Cutaneous wart Pansinusitis Bronchiectasis

BH = birth height, BW = birth weight, HF = height at first visit, HH = hair hypoplasia, ID = immunodeficiency, N/A = not available, SD = skeletal dysplasia.

* Compound heterozygous.

† Parents’ genetic data was not available, and could not be determined.

‡ Paternal allele.

§ Maternal allele.

Case 1 was diagnosed with MDWH among the CHH-AD spectrum disorders because the patient exhibited only skeletal dysplasia and lacked extraskeletal manifestations such as hair hypoplasia and immunodeficiency. However, according to a recent study, patients with MDWH features in childhood can develop late-onset extraskeletal manifestations, such as immunodeficiency or malignancy, in adulthood.^[13]^ Considering that study, the patient in case 1 should be followed up for late-onset events. MDWH is a very rare disease; only about 20 cases have been confirmed by genetic analysis, and the first case of MDWH with compound heterozygous variants (NR_003051.3:n.81G > A and NR_003051.3:n.100C > A) in Korea was reported in 2021.^[14]^

Case 2 was a classic example of CHH, characterized by skeletal dysplasia, hair hypoplasia, and immunodeficiency. Changes in hair are typically described as being easily broken and lighter in color than in their siblings.^[15]^ Eighty-eight percent of CHH patients have abnormal cellular immunity, and their clinical symptoms are generally limited to early childhood.^[2,16]^ However, some cases can progress to SCID, which is associated with an increased mortality risk. Because of the accompanying immunodeficiency, patients with CHH can suffer from recurrent respiratory infections, leading to lung structure diseases, such as bronchiectasis. Furthermore, pneumonia in the first year of life or recurrent infections in adulthood is another risk factor for early death.^[10]^ Autoimmune disease has been reported among individuals with CHH, including immune-mediated thrombocytopenia, autoimmune hemolytic anemia, enteropathy, thyroid disease, and juvenile idiopathic arthritis. The prevalence of autoimmune diseases in CHH was reported to be 10.6% in a Finnish cohort.^[17]^ The 2 patients in our report did not show any autoimmune dysfunctions.

Reportedly, the most prevalent cancers in CHH patients are non-Hodgkin lymphoma and skin cancer, particularly basal cell carcinoma.^[18]^ The typical age for a cancer diagnosis in CHH patients is 15 to 44 years, and multiple malignancies have also been reported.^[19]^ Several studies have demonstrated that inappropriate control of infectious disease is associated with the pathogenesis of certain cancers in CHH, and a correlation between warts and the development of skin cancer in CHH patients has also been reported.^[5]^

**Figure 3.**
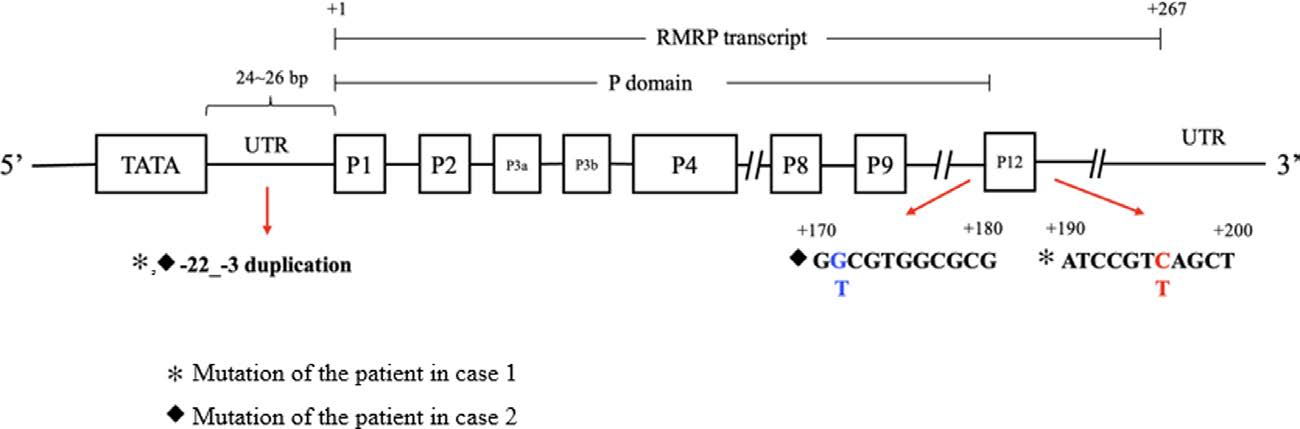
Schematic representation of the *RMRP* gene. RMRP = the RNA component of the mitochondrial RNA-processing Endoribonuclease.

Several pathogenic variants in the *RMRP* gene, insertions, and duplications between the TATA box and the transcription start site are associated with CHH.^[1]^ The genotype–phenotype correlation and underlying mechanism are not entirely understood. However, the phenotype of the CHH spectrum can be affected by whether variants are in the transcribed region or promoter region and are in the RNA-to-protein binding region or not. In addition, it has been shown that reduced cleavage of rRNA correlates with the severity of skeletal dysplasia. In contrast, reduced cleavage of mRNA correlates with milder skeletal dysplasia with additional extraskeletal features.^[1]^ We display the schematic representation of the *RMRP* gene, the sequencing outcomes for the 3 variants discovered in our cases (n.-22 -3dup; n.196C > T; and n.171G > T), and the sequencing results in Figure 3. Two previously reported pathogenic variants^[1,20–22]^ (n.-22_-3dup; n.196C > T) were identified as pathogenic variants. One variant (n.-22_-3dup) involves the duplication of 20 nucleotides in the promoter region of the *RMRP* gene, which is located between the TATA box (−33 to −25) and the transcription initiation site. Another variant (n.196C > T) is located near the P12 domain and has been reported to result in a mild to intermediate decrease in RNA cleavage activities.^[1]^ The other variant (n.171G > T) has not been reported, but a similar variant (n.171G > A) has been reported to be associated with the CHH spectrum.^[23]^ Additional functional studies are needed to confirm the pathogenicity of the n.171G > T variant.

## 4. Conclusions

Here, we present 2 Korean cases of CHH-AD with *RMRP* gene mutations, 1 of which is the first Korean patient with CHH. With skeletal dysplasia (typically metaphyseal abnormalities), the CHH-AD spectrum can present with various clinical manifestations depending on the presence or absence of extraskeletal manifestations and immunodeficiencies, which significant impact on the prognosis. This is the first Korean CHH reported, and our cases suggest that children with short stature and immunodeficiency should include CHH as a differential diagnosis. The *RMRP* gene is a noncoding, untranslated RNA gene and may not be included in the scope of next-generation sequencing or WES. An exome kit from WES performed well at capturing the targets, however, the coverage is an important matter. In diagnosing CHH, clinical and radiologic suspicion is most important, but the *RMRP* gene should also be considered in genetic testing.

## Acknowledgments

We would like to thank all individuals living with rare genetic diseases, their families, and all clinical and laboratory staff.

## Author contributions

**Conceptualization:** Ju Heon Park, Yae-Jean Kim, Ja-Hyun Jang, Min-Sun Kim, Sung Yoon Cho.

**Data curation:** Ju Heon Park, Minji Im, Yae-Jean Kim, Ja-Hyun Jang, Sae-Mi Lee, Min-Sun Kim, Sung Yoon Cho.

**Formal analysis:** Ju Heon Park, Minji Im, Yae-Jean Kim, Ja-Hyun Jang, Sae-Mi Lee, Min-Sun Kim, Sung Yoon Cho.

**Funding acquisition:** Ju Heon Park, Ja-Hyun Jang, Min-Sun Kim, Sung Yoon Cho.

**Investigation:** Ju Heon Park, Minji Im, Ja-Hyun Jang, Sae-Mi Lee, Min-Sun Kim, Sung Yoon Cho.

**Methodology:** Ju Heon Park, Minji Im, Yae-Jean Kim, Ja-Hyun Jang, Sae-Mi Lee, Min-Sun Kim, Sung Yoon Cho.

**Project administration:** Ju Heon Park, Minji Im, Yae-Jean Kim, Sae-Mi Lee, Min-Sun Kim, Sung Yoon Cho.

**Resources:** Ju Heon Park, Minji Im, Yae-Jean Kim, Ja-Hyun Jang, Sae-Mi Lee, Min-Sun Kim, Sung Yoon Cho.

**Software:** Ju Heon Park, Minji Im, Ja-Hyun Jang, Sae-Mi Lee, Sung Yoon Cho.

**Supervision:** Minji Im, Yae-Jean Kim, Ja-Hyun Jang, Sae-Mi Lee, Sung Yoon Cho.

**Validation:** Ju Heon Park, Minji Im, Yae-Jean Kim, Ja-Hyun Jang, Sae-Mi Lee, Min-Sun Kim, Sung Yoon Cho.

**Visualization:** Ju Heon Park, Minji Im, Ja-Hyun Jang, Sae-Mi Lee, Min-Sun Kim, Sung Yoon Cho.

**Writing—original draft:** Ju Heon Park, Minji Im, Yae-Jean Kim, Ja-Hyun Jang, Sae-Mi Lee, Min-Sun Kim, Sung Yoon Cho.

**Writing—review & editing:** Minji Im, Ja-Hyun Jang, Sung Yoon Cho.
